# Plasmonic Sensor Based on Dielectric Nanoprisms

**DOI:** 10.1186/s11671-017-2347-7

**Published:** 2017-11-03

**Authors:** Mahmoud H. Elshorbagy, Alexander Cuadrado, Javier Alda

**Affiliations:** 1 Optics Department, University Complutense of Madrid, Faculty of Optics and Optometry, Av. Arcos de Jalón, 118., Madrid, 28037 Spain; 2 Physics Department, Faculty of Science, Minia University, University campus, El-Minya, 61519 Egypt

**Keywords:** Optical sensor, Nano-prism, Guiding light, Plasmonic sensor, Biosensor

## Abstract

A periodic array of extruded nanoprisms is proposed to generate surface plasmon resonances for sensing applications. Nanoprisms guide and funnel light towards the metal-dielectric interface where the dielectric acts as the medium under test. The system works under normal incidence conditions and is spectrally interrogated. The performance is better than the classical Kretschmann configurations, and the values of sensitivity and figure of merit are competitive with other plasmonic sensor technologies. The geometry and the choice of materials have been made taking into account applicable fabrication constraints.

## Background

The use of surface plasmon resonances (SPR) for optical sensing gained great attention as they provide label-free devices for biomedical and material science. These sensors work with spectral or angular interrogation procedures [1–5], and some of them make use of colorimetric changes detectable by the human visual system [6, 7] The basic setup for the excitation of surface plasmon resonances is the classical Kretschmann configuration [8] where light is incident at a given angle on a thin metal sheet from a dielectric transparent prism that is in direct contact with the metal layer [9]. Otto configuration also uses a prism, but now, the metallic layer is separated from the prism by a thin space where the plasmon resonance takes place [10]. A variation to the previous classical configurations uses a hemispherical lens and a grating that couples radiation at the plasmon resonance interface [11]. The output from the Krestschmann setup depends on the wave vector matching condition that should be fulfilled for a given angle of incidence at the metal dielectric interface. This condition can be written as

1 $$ \frac{2\pi}{\lambda} n_{P} \sin \theta_{r} = \text{Re} \left[ \beta^{\text{SP}} \right] , $$

where *n*_*P*_ is is the refractive index of the prism and *β* ^SP^ is the propagation constant of the surface plasmon generated at an angle of incidence *θ*_*r*_ [12, 13]. The angle of incidence is typically quite large, and this fact sometimes limits the operational range and the operative easiness of the device. To overcome these constraints, several proposals for integrated SPR sensors have been analyzed in the literature. For example, very narrow grooves on thin metal films excite SPR under normal incidence conditions [14]. However, the very narrow width of the grooves, in the range of 3 nm, may compromise the device fabrication. A similar approach that is achieved experimentally is the excitation of SPR using narrow metallic nanocavities [15]. Another approach has been demonstrated theoretically using metallic gratings embedded in a glass substrate, obtaining spectral reflectances showing acute dips with widths or around 3 nm [16]. These approaches allow normal incidence conditions, and the interrogation method is now based on the spectral variation of the reflected light. This is why sharp spectral features are very much appreciated to improve the performance of those sensors. We have chosen spectral reflectivity to allow reading the signal from the incidence side. Optical absorption enhancement produced by plasmonic nanostructures excited at normal incident conditions also provides an alternative to the Kretschmann configuration. This approach uses absorption as a sensing parameter for photo-detection [17, 18].

In this contribution, we propose to maintain normal incidence conditions for the incoming light and make use of funneling mechanisms in dielectric structures to direct light towards the locations where SPR are generated. High-aspect ratio dielectric gratings (HARDG) have been proposed to guide light into active layers of photovoltaic cells [19]. The same concept is applicable to sensing devices redirecting light towards the metal-dielectric interface of interest. In this contribution, we propose the use of nanoprisms embedded on a dielectric substrate that is flat and adjacent to the metal-dielectric layer used for sensing through the excitation of SPR. This structure funnels the incoming radiation more efficiently, and therefore, plasmon resonances benefit from the increase in the energy reaching the plane of interest. The proposed devices perform better than similar structures and have geometrical and material arrangements that are feasible and fabricable with standard nanofabrication techniques.

## Methods

**Fig. 1:**
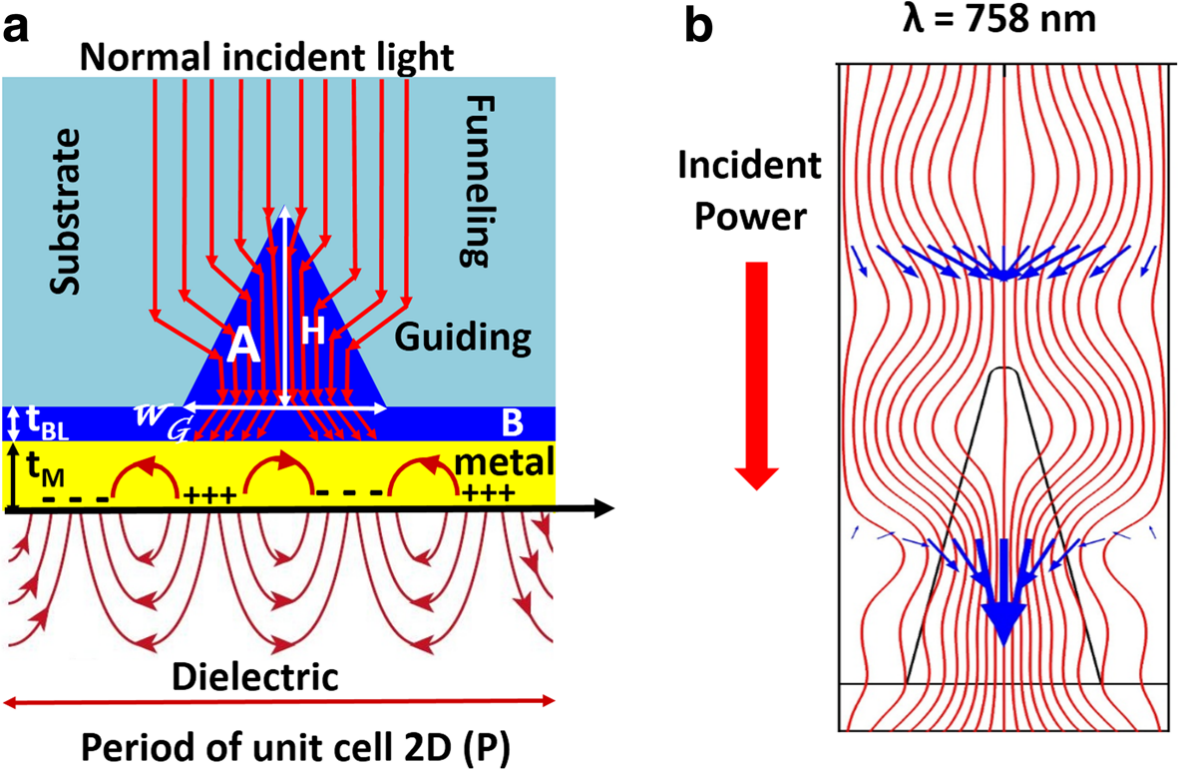
**a** Schematic diagram of the proposed structure and **b** time-averaged power flow at *λ* = 758 nm for the proposed structure without the metal layer where the funnelling mechanism is shown

The geometry of the proposed structure can be seen in Fig. 1a. Light is normally incident towards the tip of an isosceles nanoprism array. We consider a MgF_2_ substrate that can be etched, or patterned, with periodic longitudinal grooves having the desired triangular shape [20, 21]. These grooves are filled with aluminum zinc oxide (AZO). This material can be spin-coated over the nanopatterned substrate to produce a planar interface for the deposition of a metal thin film, for example, gold to assure good biocompatibility. Finally, we have considered water as the medium under test in order to mimic biosample conditions. The optical constants for the materials have been obtained from [22] for MgF_2_, [23] for AZO, and [24] for gold. This selection of materials has been guided by a first analysis of the feasibility of the device in terms of fabrication constraints. The index distribution is appropriate when considering the matching between a low index substrate (MgF_2_) and a high index buffer layer (AZO). The reliability of the optical constants is a key factor when analyzing the validity of the numerical model. A refinement of the computational model should require the characterization of the materials fabricated with the same technique and arrangement used to manufacture the devices. As far as we are analyzing the parametric optimization of the device, we are extracting the optical constants from commonly used references for each material. In the case of gold, the values from reference [24] have been widely used in the literature for the analysis of similar devices [1, 13, 25].

The proposed material arrangement enhances the funneling effect already observed in some HARDG. The funneling and guiding effects in HARDG couples radiation towards the thin metal film where the SPR is generated.

A preliminary analysis considers a TM plane wave normally incident from the substrate side on the structure, without incorporating the metal layer. The amplitude of the incident electric field is 1 V/m. The results for this structure (see Fig. 1b) show how light is funneled and guided through the prism reaching the region where the metal-dielectric interface generates SPR. The field available at this region is stronger than that of the classical Kretschmann setup. This configuration shows a very strong plasmonic resonance at some specific wavelengths determined by the geometrical parameters of the structure. Additionally, the geometry of the device and the choice of materials are of great importance to properly operate the device. The geometry of the system is determined by the thicknesses of the buffer and metal layers, *t*_BL_ and *t*_M_, and by the parameters defining the nanoprism (width and height, *w*_*G*_ and *H*), and its spatial periodicity, *P*. The three-dimensional shape of the nanoprism is extruded from a two-dimensional design (see Fig. 1a). The prism region is divided into two portions, A and B, defining the groove array and the plane-parallel buffer layer. These two regions can be fabricated with the same material or using two materials. These two configurations will produce different spectral behaviors.

The analysis of the performance of this device is made by a computational electromagnetism package (COMSOL Multiphysics) based on a finite element method. The COMSOL model has been positively checked by evaluating the behavior of the classical Kretschmann configuration and comparing the numerical results against the analytical solution [12]. The results obtained from the computation have been used to optimize the design with two main goals: to increase the field amplitude at the location where SPR are generated (metal-water interface) and to decrease the width of the reflectance dip associated with the resonance. This resonance is parameterized by the full-width-at-half-maximum (FWHM) of the reflectance.

Actually, most of the SPR sensors work as refractometers because they sense very well the change in the index of refraction of the medium under analysis. In this case, sensitivity is defined as [13]:

2 $$ S_{B}= \frac{\Delta \lambda}{\Delta n}   $$

that describes the shift of the spectral location of the minimum of reflectance, *Δ* *λ*, when the index of refraction changes, *Δ* *n*. Sensitivity is given as nm/RIU, where RIU denotes refractive index units. Another parameter to compare different sensor technologies is the figure of merit (FOM) that is defined as

3 $$ \text{FOM} = \frac{S_{B}}{\text{FWHM}}.   $$

This parameter is the ratio of sensitivity to the spectral width of the reflectance dip, and it is given as 1/RIU. This figure of merit already considers the capability of a given system to sense a given change in the location of the minimum of reflectance.

The evaluation of the field enhancement at the analyte location, and the reflectance FWHM at the peak, takes a quite long time using dedicated computers. This fact makes multidimensional optimization harder to solve. Besides, it would need the definition of a merit function combining properly the performance parameters. Then, we choose to take one parameter at the time to optimize the device. This strategy is well suited to understand how each geometrical parameter changes the overall performance of the device. Additionally, by monitoring and optimizing the field enhancement and the FWHM of the spectral reflectance, we also obtain higher values for the sensitivity and FOM. After optimization, we found that the geometrical parameters producing a better response are *t*_BL_ = 100 nm, *t*_M_ = 30 nm, *w*_*G*_ = 325 nm, and *H* = 700 nm and a periodicity of *P* = 550 nm. These values have been obtained taking into account the fabrication constraints. This is why we have considered a step of 25 nm between successive values included in the optimization. We have also avoided the use of ultra-thin or ultra-thick layers that could compromise the feasibility of the device.

**Fig. 2:**
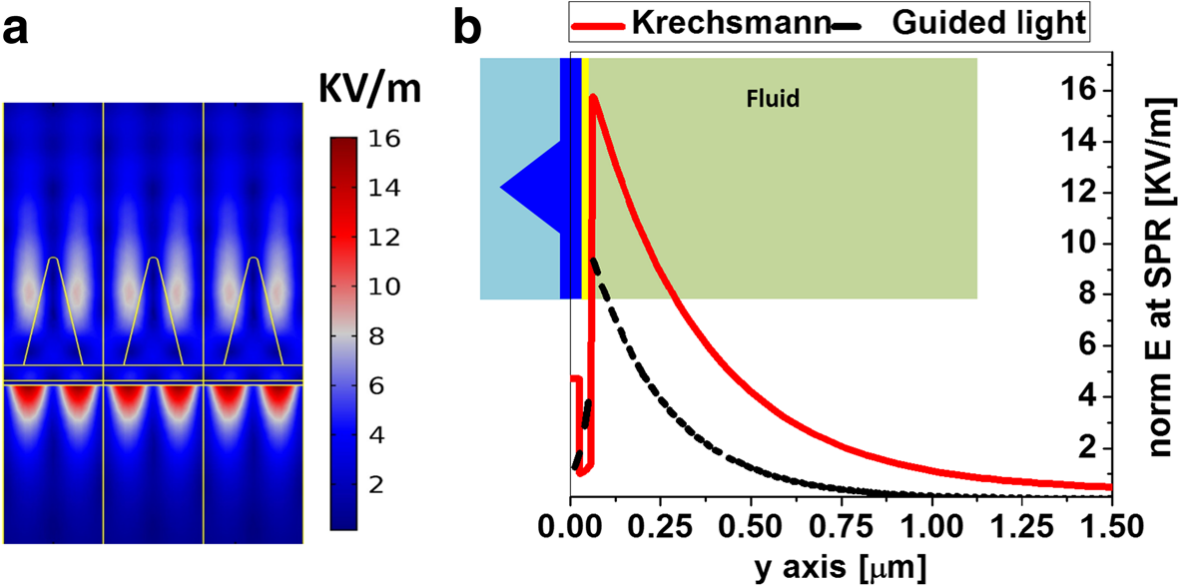
**a** Map of the modulus of the electric field at *λ* = 758 nm for an input electric field amplitude of 1 V/m and polarized as a TM mode (electric field parallel to the map). **b** Profile of the electric field magnitude along the direction of propagation for the Krestchmann configuration (*black dashed line*) and for the nanoprism device (*red solid line*)

Figure 2a shows a map of the modulus of the electric field at the resonance wavelength *λ* = 758 nm for the proposed structure when an incoming wavefront having an amplitude of 1 V/m illuminates the system. The polarization corresponds to a TM mode. The wavelength used for optimization is chosen arbitrarily and, if necessary, can be shifted by changing the period parameter, *P*. To compare our results with those obtained from the classical Kretschmann configuration, we evaluate its performance using the same wavelength, *λ* = 758 nm, to illuminate the prism. Then, we calculate the angular dependence of the reflectivity to obtain the incidence angle at which the resonance takes place for the Kretschmann prism, which is 66.28° for BK7 glass/Au [50 nm]/water. The normalized electric fields at resonance for the classical Kretschmann setup and that of the nanoprism configuration are presented in Fig. 2b. They show a significant enhancement of the evanescent field in the analyte medium due to the focusing effects (funneling and guiding) produced by the nanoprism. This enhancement is larger in the proposed device that works under normal incidence conditions. Besides the field enhancement obtained with the nanoprism device with respect to the Krestchmann configuration, we can see that the plasmon resonance propagates within the medium under test along an estimated depth of 180 and 300 nm for Kretschmann setup and our proposal, respectively. Therefore, the interaction volume of the proposed nanoprism structure is larger than in the Krestchmann setup.

**Fig. 3:**
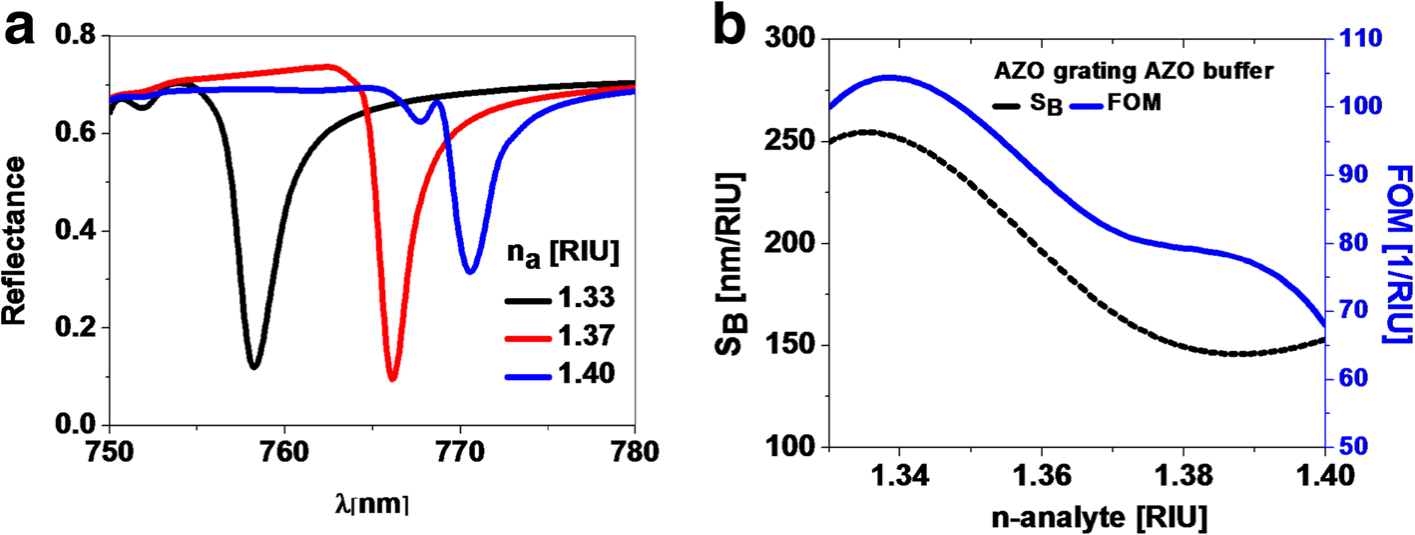
**a** Spectral reflectance for an optimum design that uses AZO as the buffer layer as a function of the index of refraction of the medium under test. The sharpness of the resonance peak degrades as the index of refraction increases. **b** Sensitivity (left axis and black dashed line) and figure of merit (right axis and blue solid line) as a function of the index of refraction of the medium under test

The values of sensitivity and FOM (Eqs. 2 and 3) are evaluated from the spectral behavior of the reflectance when changing the index of refraction of the medium under test. In Fig. 3a, we have plotted several reflectance curves for different values of the index of refraction of the analyte. Figure 3a shows a degradation in the sharpness of the minimum when the index of refraction of the analyte becomes closer to the buffer layer index. In this situation, which involves a very thin metal film, reflectance becomes smaller because the difference in the index of refraction diminishes. The maximum values for *S*_*B*_ and FOM obtained from Fig. 3b are 250 [nm/RIU] and 100 [1/RIU] respectively. These values are higher than the previously reported results for classical Kretschmann configurations [26–30]. However, these values for both *S*_*B*_ and FOM are not constant when changing the refractive index of the analyte [30–33].

## Results and Discussions

In the previous optimization process, we paid attention to the geometry of the device. Now, we analyze how a different choice of materials can improve the performance of the device. To do that, we distinguish between the nanoprism region and the plane-parallel layer separating the nanoprism from the metallic deposition (portions A and B in Fig. 1a). Then, the nanoprism material is still made of AZO to preserve the funneling characteristics and easiness of fabrication using spin-coating techniques. In region B, we replace AZO by GaP (optical constants obtained from [34]). This change solves the degradation of the sharpness of the reflectance peak when moving to a higher index (see Fig. 3a). When analyzing the final optimized design, we will resume this comparison. This behavior is well appreciated to improve the stability and reliability of the sensor.

**Fig. 4:**
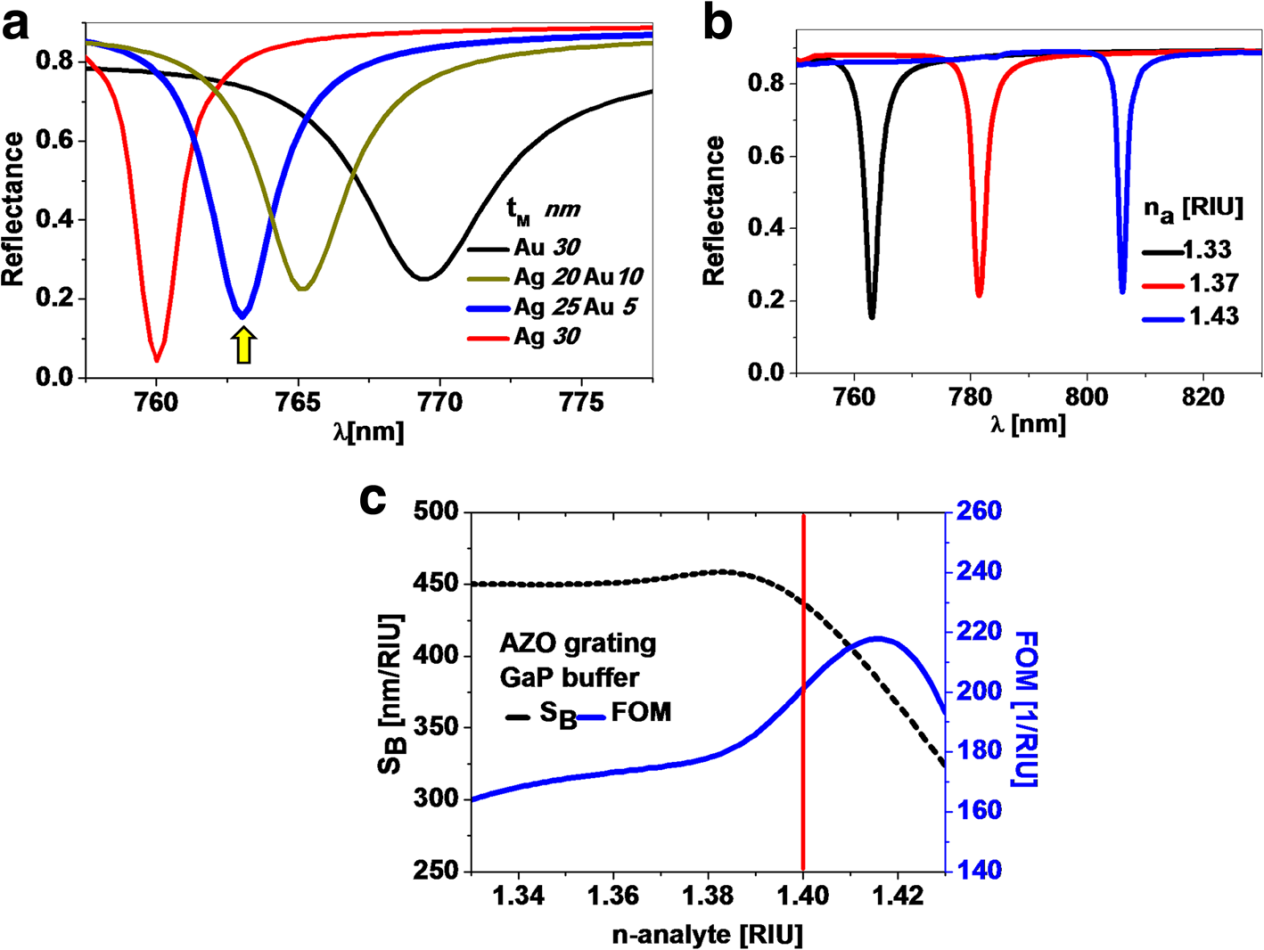
**a** Spectral reflectance for single-metal 30-nm-thick layer made of gold (black) or silver (red), and for bi-metallic layer for two thicknesses combinations (blue and green). The yellow arrow selects the response for the optimum arrangement (25 nm-Ag / 5 nm-Au). **b** Spectral reflectivities of the optimum device that use a GaP buffer layer. The peaks show a similar sharpness for three different values of the index of refraction. **c** Sensitivity (left axis and black dashed line) and FOM (right axis and blue solid line) of the optimized sensor for an extended range of refractive index. The vertical line denotes the limit analyzed in the previous design where the buffer layer was made of AZO and the metallic layer was made of gold

The next material to analyze is the metal used for the generation of SPR. The choice of gold is based on its good biocompatibility. However, silver (optical constants obtained from [24]) is better suited to generate a stronger SPR. To take advantages of both characteristics, we propose a dual successive deposition to fabricate a bi-metal layer made of silver and gold. In Fig. 4a, we have plotted four possible options for the metallic layer. The reflectance of silver (red line in Fig. 4a) shows a sharper, narrower, and deeper reflectance peak than gold (black line in Fig. 4a). The peak for the silver is located at a shorter wavelength than the resonance for a gold metallic layer. The spectral reflectance for the combination of these metals in the bilayer structure lies in between the two single-metal options, showing a better resonance as the gold layer becomes thinner. An optimum solution is a bilayer made of 25-nm-thick silver coated with 5-nm-thick gold. This solution combines both metals with thicknesses in the range of the fabrication technology.

For the optimum case of a bi-metal layer considered previously, we have plotted in Fig. 4b the spectral response for several values of the index of refraction. When comparing the spectral reflectances in Figs. 3a and 4b, we can also check how the sharpness of the spectral peak is maintained for a larger range in the index of refraction of the analyte. The reason for this improvement is the use of GaP in the fabrication of the buffer layer of the device. Figure 4c contains the values of sensitiviy and FOM for the optimized device that contains a bimetallic layer (25 nm silver/5 nm gold) and a GaP buffer layer. These values are higher than those presented in Fig. 3b where we had a single-metal gold layer and an AZO buffer layer. Figure 4c includes a vertical red line that signals the upper limit in the index of refraction where the design analyzed in Fig. 3 begins to degrade the sharpness of the spectral reflectance peak. The optimum structure has a maximum *S*_*B*_=450 nm/RIU, which is stable over a wide range of refractive index changes and corresponds to a FOM ranging from 160 to 220 1/RIU.

These values are better than some recent proposals that use graphene [28, 30, 35], silicon nanostructures [27], dielectric or metallic gratings [26, 29], oxide films [36], and metallic nanoprisms (gold coated over silver nanoprisms) [37]. When not working at normal incident, some other plasmonic structures, as the gold mushrooms, show a higher sensitivity but a lower FOM [38].

## Conclusions

This contribution presents a dielectric nanoprism extruded geometry that increases the available power to generate SPR at the sensing surface. Therefore, The SPR extends deeper within the analyte and, consequently, it increases its interaction volume. This characteristic should lower the limit of the detection of the system. The device works under normal incidence conditions. This makes possible an easier integration of the illumination and interrogation system, for example, placing the sensor at the tip of an optical fiber. The performance of the system is better than the previously reported results in this field. Sensitivity shows a plateau of around 450 nm/RIU for a large range in the index of refraction (from 1.33 to 1.39). The figure of merit, FOM, is also large and has a minimum value of 160 and a maximum of 220 1/RIU in the whole range of index of refraction between 1.33 and 1.43. To obtain these figures in performance, the design has been optimized by changing its geometrical parameters and the material choice. We have also considered materials that can be incorporated in a fabrication strategy involving spin coating. This allows the planarization of the device and does not interfere with the refraction index matching conditions. In this optimization, we have always keep in mind the feasibility of the fabrication, avoiding very narrow features that could compromise the device. The optimization in terms of the material choice has substituted AZO by GaP at the buffer layer to extend the range in the index of refraction from 1.40 to 1.43. Also, we have dimensioned a silver-gold bimetallic layer that takes advantage of the good plasmonic response of silver and the biocompatibility of gold. The nanoprism structure presented here improves operational easiness, allowing a normal incidence setup, and can be used for biomedical, environmental, or industrial applications involving liquids.
